# Stability in biosensors derived from domain map analysis of bibliometric data

**DOI:** 10.3389/abp.2024.12196

**Published:** 2024-01-23

**Authors:** Aleksandra Klos-Witkowska, Vasyl Martsenyuk

**Affiliations:** Department of Computer Science and Automatics, University of Bielsko-Biala, Bielsko-Biala, Poland

**Keywords:** biosensor, stability, clusters, trends, leaders, scientometric database

## Abstract

In the presented work, advanced methods of analysis and visualization were used to compile trends and patterns in the scientific literature. The most relevant information for the stability of biosensors was selected on the basis of clusters constructed on the basis of keywords. The most significant publications in the clusters appearing over time were analyzed. The most explosive publications were identified, i.e., those that have had the greatest impact on science in the area of the subject under study. The scientific trend in the development of biosensor stability was determined on the basis of the most frequently cited words in recent publications. A map of cooperation and networking between countries in the field of interest of the above topic was presented. Leaders were identified by country of origin.

**Systematic Review Registration:** https://ubb.edu.pl.

## Introduction

The number of scientific publications increases every year with the development of technology and the progress of civilization. Therefore, the need for a fast and accurate analysis of the literature to keep track of the development of existing research topics and emerging news in the world of science was naturally born. Previously used scientific analysis based on web reviews or studying scientific databases such as Web of Science, Scopus, PubMed allowed only retrospective analysis, i.e., chronological, recollective analysis without the ability to trace connections. Domain map analysis means the use of graph network representations of the source analysis, including the analysis of the relationships between data with the help of the choice and appropriate distance measure, clustering, mapping the relationships into graph nodes and edges. It allows us to determine the most significant resources analyzed temporal development of the area determined tendencies (Liu et al., 2020; Geng et al., 2023).

One of the most popular tools for bibliometric analysis of domain maps is CiteSpace, which is based on data mining, a process involving data extraction, pattern analysis and sorting. It is a process that can be compared to mineral processing, where the mineral must first be extracted, then cleaned and finally sorted. Data mining enables rapid predictions and classifications to be made and facilitates decision making.

CiteSpace is a Java based application designed to analyze and visualize trends and patterns in the scientific literature to present the structure and distribution of scientific knowledge. It focuses on finding critical points in the development of a field or region, especially intellectual turning points (Xu et al., 2022; Gao et al., 2023; Zhao et al., 2023). In presented papers we used CiteSpace to group keywords and highlight results related to biosensors stability.

The analysis is based on the theoretical trend (intellectual) and the experimental trend. The intellectual trend is based on articles presenting research findings or a scientific point of view, while the experiential trend [as conceptualized by Price (1965)] is based on collections of cited articles.

The functions of the CiteSpace application are based on three central concepts:

1. The Kaliberg crack detection algorithm (Kleinberg, 2002), which is used to identify test concepts.
2. The Freman intercentricity matrix, used to highlight potential key points
3. The Heterogeneous Network, used to map between the intellectual base and the experimental front.

Mapping can be used to identify the nature of the research front, highlight specializations and identify emerging trends over time.

The CiteSpace analysis are based on select single words or phrases of up to four keywords from titles, abstracts and article descriptions based on articles by the method LSI (Latent Semantic Indexing) related to biosensors stability.

The research is based on a surge in citation frequency. Analysis by cluster view and by temporal variation is possible. The CiteSpace tool makes it possible to follow in real time the development of new fields of knowledge and the links in the existing field of knowledge (between institutions and countries), it also gives the possibility to see the history of the emergence of a scientific question and the impact of the main discoveries on the final view. The present article analyses the stability of biosensors on the basis of experimental studies. The analysis was carried out using the CiteSpace 6.2. R4 tool, which selects the most important articles that cause the scientific development of the topic, based on the keywords that appear most frequently in the titles and abstracts. The novelty of the article is: 1) the presentation of the latest trends, the indication of the direction of research in the field of stability of biosensors, based on an advanced analysis using the CiteSpace tool, 2) the identification of the scientific leaders dealing with the studied issue, and 3) the presentation of the mutual network of scientific links between research groups coming from different countries.

## Methods

### Sources of data

In this paper we used the Web of Science (WoS) database as a data source and through advanced search (TS= biosensor) we obtained 86,814 papers in the range time (01.01.1974–01.07.2023).

**FIGURE 1:**
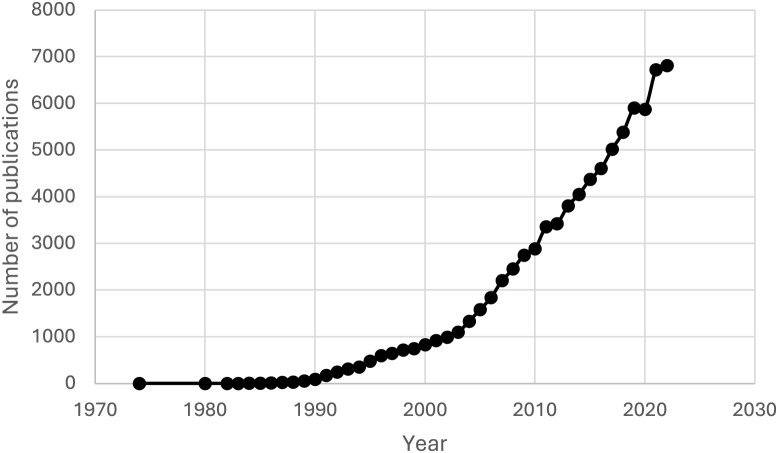
Number of publication in the biosensor field from 1974 to 2022.

Figure 1 shows the number of publications in the field of biosensors from 1974 to 2022. We have chosen 2022 as the upper limit of the time frame because this is the last full calendar year in the WoS database (2023—not yet over). The graph shows a clear increase in publications over time, indicating a growing interest in biosensors, most likely related to the increase in environmental pollution (Pohrebennyk et al., 2018) and the widening range of biosensor applications [medicine (Grabowska et al., 2014), environmental protection (Klos-Witkowska, 2016), food industry (Dirpan et al., 2023) and the drive to improve existing devices to achieve even better detection quality (Laad and Ghule, 2022)]. It is worth noting the slight decrease in the number of papers published in 2020 (5,875 papers) compared to 2019 (5,902 papers), perhaps due to the impact of the COVID-19 pandemic, which was felt all over the world.

Current research is carried out in two parallel streams: experimental and theoretical.

In the experimental stream, work focuses on improving the quality of detection and the stability of the device (Klos-Witkowska and Kajstura, 2020), while the theoretical stream revolves around the development of mathematical models (Martsenyuk et al., 2021b), often using already existing solutions for this purpose (Martsenyuk et al., 2021a), also recently, the interest of scientists in combining biosensors with artificial intelligence has been noted. An extremely interesting issue is the problem of stability in biosensors, which is a very important problem not only from a scientific point of view, but also from a commercial point of view. The stability of biosensors directly translates into the longevity of use and the ability to operate the device. Therefore, following the latest scientific trends, the issue of biosensor stability was chosen to be analysed in biosensor development based on advanced analytics using CiteSpace 6.2.R4;

(1) Identifying the most important (so called “explosive articles”) that generated the most interest among researchers based on their citability.
(2) The most relevant keywords (most cited) in the titles and abstracts were identified and clustered, i.e., grouped according to their relevance.
(3) The most important clusters were described.
(4) The keywords with the highest number of citations on a time scale were visualised.
(5) Identified scientific leaders working on stability issues in the field of biosensors and showed the network of scientific links between research groups from different countries.

### Analytical method characteristics

We conducted a literature search using the following search strategy using Web of Science Advanced Search: Publication dates were from 01.01.1974 to 01.07.2023 and search criteria were: for biosensor stability (experimental research): [TS=(biosensor)] AND [TS=(stability)] AND [TS=(empirical)] OR [TS=(experimental)] in the Web of Science Core Collection.

We created visual graphs of keyword co-occurrence, keyword clustering and scientific collaboration using CiteSpace 6.2 R4 software. We investigated the current status and trends in the stability of biosensors.

## Results

### Biosensor stability

Biosensors are devices that are susceptible to ageing; this phenomenon can be characterised as a decrease in signal over time.

Stability of biosensors is critical for commercial success as biosensors are now used in an increasing number of different applications. Characterisation of stability in terms of shelf life, reusability and the ability to be used continuously is inadequate, although much work has been done in this area. Stability is very important as it is a major factor affecting the operation of the device.

The mechanisms of biosensor ageing are complex and still poorly understood. However, it is known that the loss of stability of a biosensor is the sum of all changes affecting both the biological material used: for example: enzymes (Fernandez-Lopez et al., 2017), antibodies (Oyetayo et al., 2017), as well as the signal mediator (Ricci et al., 2003; Malinauskas et al., 2004) and the binding material of complexes in the matrix (Panjan et al., 2017).

**FIGURE 2:**
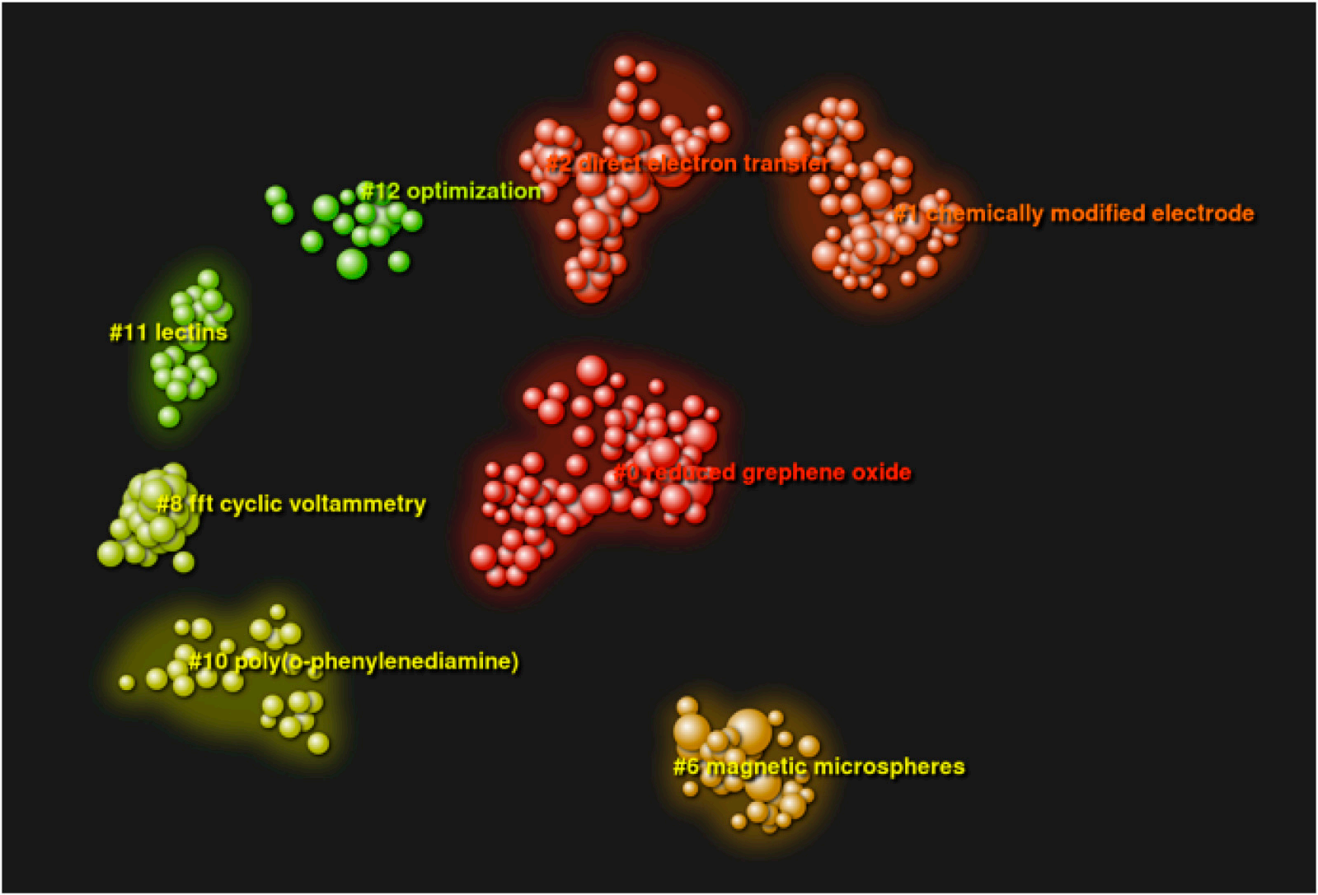
Keyword clustering analysis network.

The analysis presented below illustrates the current state of knowledge and trends in biosensor stability research. As shown in Figure 2, the efforts of researchers have focused on chemically modified electrodes, reduced graphene oxide, direct electron transfer, magnetic microspheres, FFT cyclic voltammetry, poly(o-phenylenediamine), LECTIMS and optimization. The analysis presented was carried out on the basis of the most frequently occurring keywords. These were used to create the clusters shown in Figure 2.

The larger the cluster area, the more frequent the occurrence of the keyword. In the analysis carried out, the keywords are also the names of each of the most relevant clusters, those shown in Figure 2.

Thus, it can be seen that the keyword for cluster #1 (#1) is chemically modified electrode, similarly for the others: #0 reduced graphene oxide, #2 direct electron transfer, #6 magnetic microspheres, #8 fft cyclic voltameters, #10 poly(o-phenylenediamine), #11 lectins, #12 optimisation.

The dots in each cluster represent publications; the names of the most prominent can also be seen in the diagram.

The CiteSpace analysis allows us to select the most significant sentences from the publications contained in the clusters.

Thus, cluster: #0 “reduced graphene oxide” was formed on the basis of 495 publications, of which the publications (Bai et al., 2012; Dong et al., 2012; Wei et al., 2012; Xu et al., 2012; Bai et al., 2013; Wang et al., 2013; Nalini et al., 2014; Zhou et al., 2014; Li et al., 2021) are considered to be the most significant, where one can find information on the application of gold nanoparticles and the development of an amperometric biosensor based on gold nanoparticles decorated with graphene nanoparticles for the detection of clenbuterol. Also of interest are electrochemical biosensors based on hemin-modified graphene nanoplatelets for the determination of l-tyrosine levels. Au nanoparticles were used as a stabilizer.

In cluster #1 “chemically modified electrode”; among the 243 publications forming it, the publications (Arkhypova et al., 2003; Gamella et al., 2006; Mu, 2006; Rahman et al., 2006; Wang and Zhang, 2006; Du et al., 2007; Fan et al., 2007; Fanjul-Bolado et al., 2007; Di Fusco et al., 2010; Chalikian, 2016) are highlighted. The main information in this cluster concerns: the optimization of the biosensor design, as well as the influence of experimental variables such as pH, working potential and temperature on the sensor response. The research also focused on improving the analytical characteristics of the fabricated biosensor, studies of direct electron transfer reactions, optimisation of experimental conditions and substrate affinity based on the Michaelis-Menten constant. pH, working potential and temperature are known as stabilizing factors.

Cluster #2 “direct electron transfer” consists of 460 publications. The ten most important publications are Lai et al. (2008), Zhang et al. (2008), Hsu et al. (2009), Kong et al. (2009), Nenkova et al. (2009), Zhang et al. (2009), Zhu et al. (2009), Liu et al. (2010), Lu et al. (2010), and Garay (2015). They contain information on factors influencing electron transfer. These include the results of studies describing the effect of pH, the preparation of new magnetic microspheres coated with chitosan by modifying magnetic carbon-coated iron nanoparticles. The effect of gold nanoparticles on electron transfer was also investigated.

Cluster #6 “magnetic microspheres” consists of 215 papers. The most important of these are papers (Liu et al., 2005; Tan et al., 2005; Shan et al., 2006; Fan et al., 2007; Tong et al., 2007; Liu et al., 2010; Zhen et al., 2011; Heli and Yadegari, 2014; Ltaïef et al., 2017; Villani et al., 2018) devoted to the preparation of new magnetic microspheres, control of sensor potential, detection limits and response speed from the point of view of biosensor stability.

There are 62 publications in cluster #8 “fft cyclic voltameters.” The most important are (Norouzi et al., 2010a; Norouzi et al., 2010b; Norouzi et al., 2010c; Marinov et al., 2010; Bohnenberger and Schmid, 2014; Cheraghi et al., 2017; Narwal et al., 2017; Stepashkin et al., 2018; de Oliveira et al., 2023; Kyomuhimbo et al., 2023). The key information is the application of this method to the study of: pyruvate oxidase, multiwalled carbon nanotubes immobilized on the surface of a glassy carbon electrode by means of a polymer layer of Nafion, the effect of individual components of an enzyme mixture containing gold nanoparticles, acetylcholinesterase, bovine serum albumin and glutaraldehyde on the current output of constructed acetylthiocholine biosensors. The research also included a biosensor for the detection and quantification of organophosphorus pesticides. fft cyclic voltameters allow us to investigate the stability of biosensors.

Cluster #10 “Poly(o-phenylenediamine)” was formed by 130 publications, of which the ten most important are publications (Ahammad et al., 2011; Devi et al., 2013; Zhang et al., 2014; Dervisevic et al., 2015a; Baytak et al., 2015; Dervisevic et al., 2015b; Chen et al., 2015; Tan et al., 2015; Wang et al., 2015), while clusters #11 “Lectins” and #12 “Optimization” were formed by 65 and 163 publications, respectively. The most important for cluster #11 are Bai and Shiu (2014), Gholivand et al. (2014), Oliveira et al. (2014), Ribeiro et al. (2014), Ghanbari et al. (2019), Bravo et al. (2022), Jalalvand (2022), Tvorynska et al. (2022), Zalpour et al. (2022), Ramesh et al. (2023), while for cluster 12 (Hsu et al., 2009; Kong et al., 2009; Liu et al., 2011a; Liu et al., 2011a; Liu et al., 2011b; Pasahan et al., 2011; Yang HC et al., 2011; Yang WY et al., 2011; Makhmudiyarova et al., 2015; Rakhi et al., 2016) with information on operational stability as well as sensitivity, conductivity, detection limits, response speed, detection range, calculation of the Michaelis-Menten constant, response gain.

**FIGURE 3:**
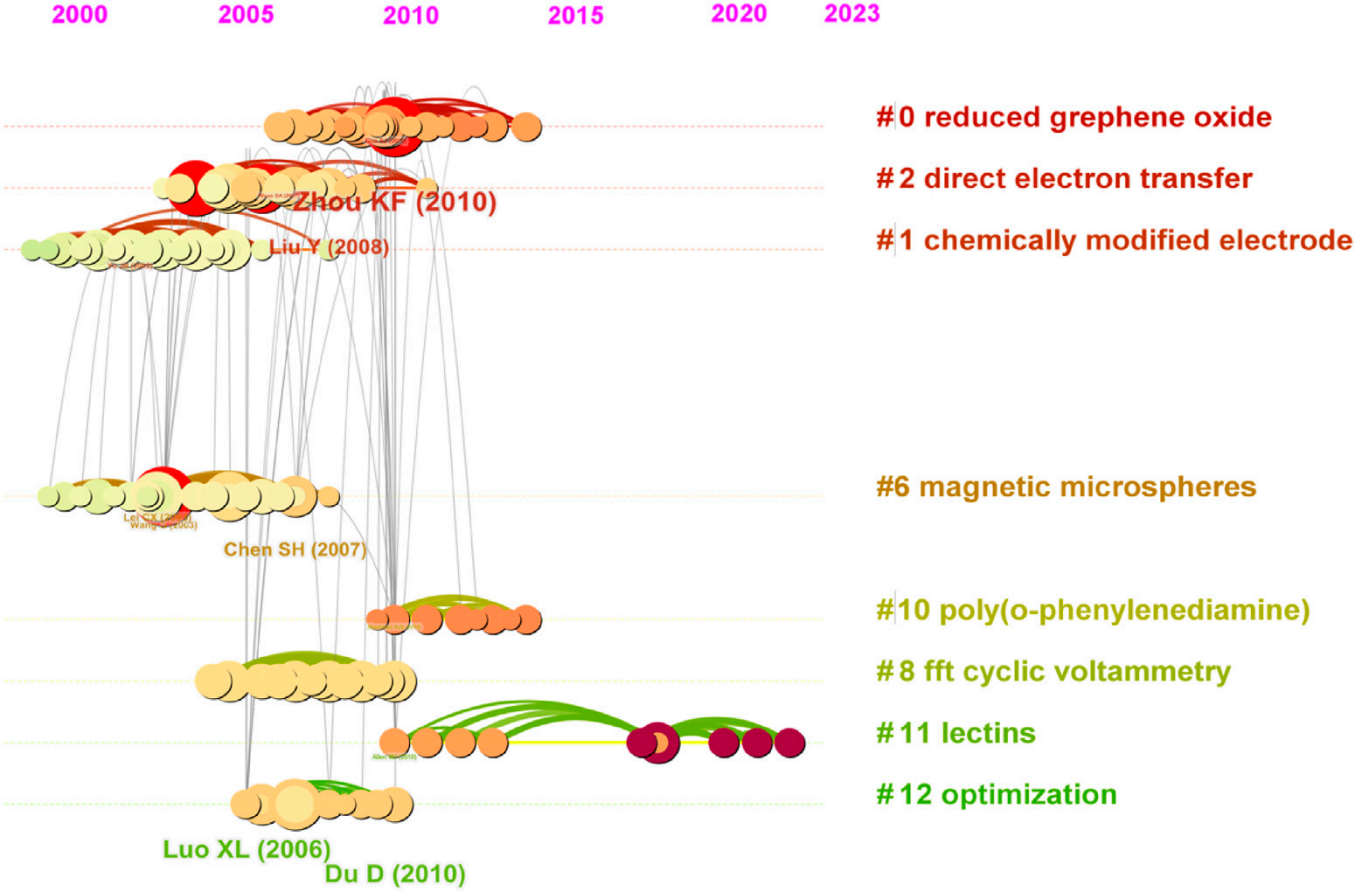
Keyword clustering analysis network in timescale.

Diagram 3 (Figure 3) shows an analysis of the most important publications in the cluster that emerged over time. The red circles indicate the most explosive publications, i.e., those that have had the greatest impact on science. These publications were selected based on the highest number of citations of words (extracted from titles, abstracts, descriptors, and identifiers of bibliographic records) found in these publications in the presented time scale.

**FIGURE 4:**
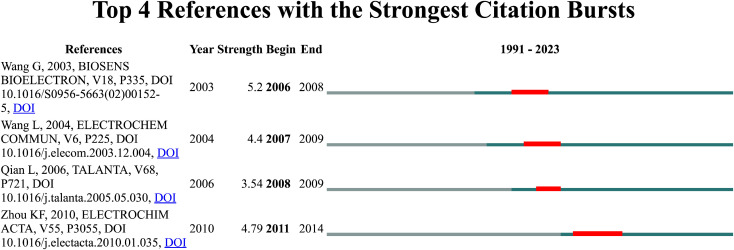
Top 4 references with the strongest citation burst.

The time scale in the graph makes it possible to determine when the explosive publication appeared. A detailed analysis is shown in Figure 4.

Thus, in the context of the stability of biosensors, it can be seen that in 2003 there was a publication (Wang et al., 2003, 267 citations) dedicated to the amperometric biosensor of hydrogen peroxide with a sol-gel/chitosan layer as immobilization matrix, which the authors describe as the development of a new hybrid sol-gel/organic composite material based on the cross-linking of the natural polymer chitosan with (3-aoryloxypropyl) dimethoxymethylsilane. This material was used to fabricate biosensors for H_2_O_2_ amperometry.

A composite membrane was used to immobilize horseradish peroxidase (HRP) on a gold disc electrode. The properties of the sol-gel/chitosan and sol-gel/chitosan-HRP layers were thoroughly characterized by atomic force and Fourier transform infrared microscopy. Using fluorescent tracers, the protein density in the sol-gel/chitosan was calculated to be 3.14 × 10^12^ molecules cm^−2^. The developed biosensor had a fast response in less than 2 s with a linear range of 5.0 × 10^−9^–1.0 × 10^−7^ mol L^−1^ and a detection limit of 2 × 10^−9^ mol. L^−1^. The reaction showed a typical Michaelis-Menten mechanism. The activation energy of the enzymatic reaction was calculated to be 8.22 kJ mol^−1^. The biosensor retained about 75% of its initial activity after about 60 days of storage in phosphate buffer at 4°C.

This was followed in 2004 by another publication (Wang and Wang, 2004, 415 citations) on a novel hydrogen peroxide sensor based on horseradish peroxidase immobilized on a colloidal Au-modified ITO electrode. The authors described the development of a novel method to fabricate a hydrogen peroxide sensor by immobilizing horseradish peroxidase (HRP) on a colloidal Au-modified conductive ITO glass substrate. The purified glass substrate was first modified with (3-aminopropyl) trimethoxysilane (APTMS) to provide an interface for the deposition of colloidal Au. Next, 15 nm colloidal Au particles were chemisorbed onto the amine groups of APTMS. Finally, HRP was adsorbed onto the surface of colloidal Au. The immobilized HRP showed an excellent electrocatalytic response to hydrogen peroxide reduction. The performance and factors affecting the obtained biosensor were investigated in detail. The obtained biosensor showed a fast amperometric response (within 5 s) to H_2_O_2_. The detection limit of the biosensor was 8.0 μmol L^−1^ and the linear range was from 20.0 μmol L^−1^ to 8.0 mmol L^−1^. In addition, the obtained biosensor showed high sensitivity, good reproducibility and long-term stability.

This is an article that is particularly important for the development of the issue related to the stability of biosensors. Despite the fact that the topic of biosensor stability is developing rapidly. An analysis conducted with the help of CiteSpace showed that no explosive articles have appeared in the last 5 years, which formed the basis for further scientific issues related to the topic of stability.

The next landmark publication was a paper (Qian and Yang, 2006, 284 citations) describing a composite layer of carbon nanotubes and chitosan for the fabrication of an amperometric hydrogen peroxide biosensor. The paper described the development of a novel amperometric hydrogen peroxide biosensor based on the cross-linking of horseradish peroxidase (HRP) with glutaraldehyde with multi-walled carbon nanotubes/chitosan (MWNTs/chitosan) coated in a composite layer on a glassy carbon electrode. The MWNTs were first dissolved in a chitosan solution. The morphology of the MWNT/chitosan composite layer was then characterised by field emission scanning electron microscopy. The results showed that the MWNTs were well soluble in chitosan and that robust layers could be formed on their surface. HRP was cross-linked with MWNTs/chitosan by glutaraldehyde to prepare a hydrogen peroxide biosensor. The enzyme electrode showed excellent electrocatalytic activity and fast response for H_2_O_2_ in the absence of mediator. The linear detection range of H_2_O_2_ (applied potential: −0.2 V) was from 1.67 × 10^−5^ to 7.40 × 10^−4^ M with a correction factor of 0.998. The biosensor showed good reproducibility and stability in the determination of H_2_O_2_. There was no interference from ascorbic acid, glucose, citric acid and lactic acid.

The last of the four most explosive scientific publications was a paper (Zhou et al., 2010, 441 citations) on a novel hydrogen peroxide biosensor based on Au-graphene-HRP-chitosan biocomposites. The paper used graphene very effectively to construct an H_2_O_2_ biosensor. Graphene and horseradish peroxidase (HRP) were co-immobilised in a biocompatible chitosan (CS) polymer, and then a glassy carbon electrode (GCE) was modified with the biocomposite, followed by electrodeposition of Au nanoparticles on the surface to form an Au/Graphene/HRP/CS/GCE layer. Cyclic voltammetry showed that direct electron transfer of HRP was realized, and the biosensor had excellent performance in terms of electrocatalytic reduction towards H_2_O_2_. The biosensor exhibited high sensitivity and speed. In addition, the biosensor showed good reproducibility and long-term stability.

As can be seen from the most explosive publications, the stability of biosensors is primarily related to the generation of optimal substrates (i.e., receptor layers).

**FIGURE 5:**
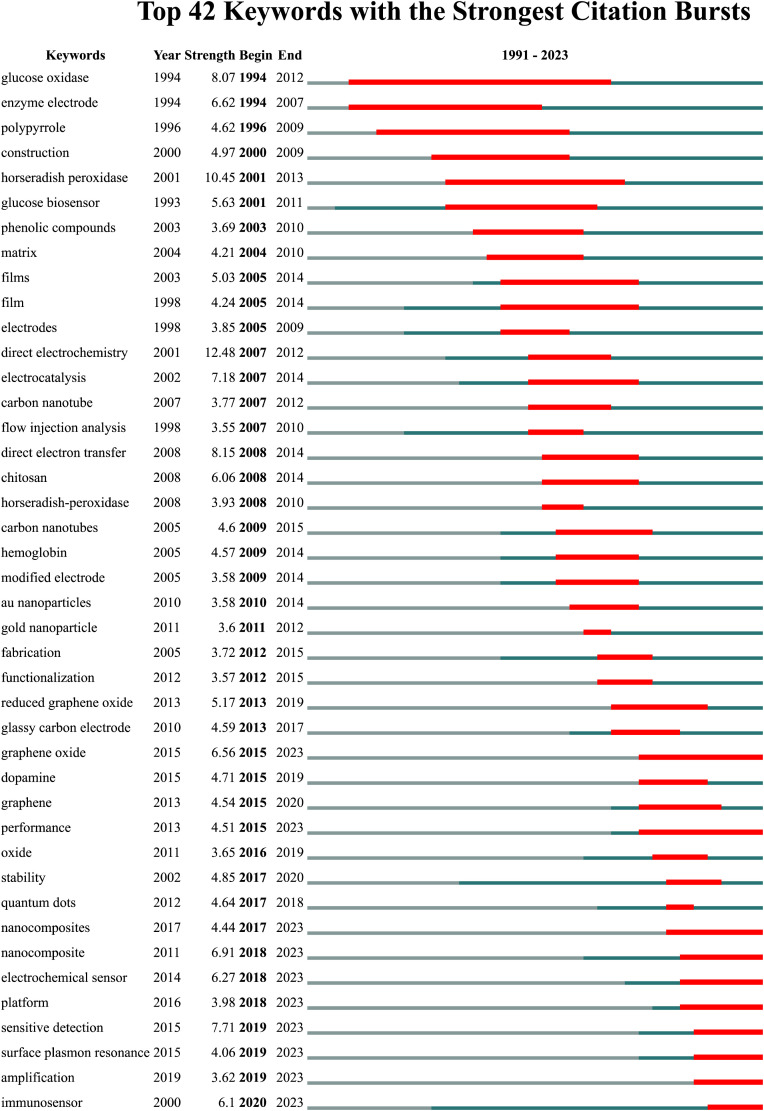
Top 42 keywords with the strongest citation bursts.

The range of words considered to be the most explosive, i.e., those that appear most frequently in citations, is shown in Figure 5.

CiteSpace 6.2 R.4 software was used to highlight keywords. The tool used performed a keyword analysis, i.e., it extracted the most frequently cited words. The graph shows a time scale indicating the period of the highest citation of a given keyword. It can be seen that although the analysis covered works from 01.01.1974 to 01.07.2023, the graph shows the period from 1991 to 2023. It can be seen that the dominant keyword is “glucose oxidase,” this term has also been cited the longest, as can be seen in the figure. A similar reasoning can be applied to the other words in Figure 5.

Analyzing the entries below, the terms that deserve special attention are those that remain in quotation marks to this day. Among them we can distinguish: “ Graphene oxide,” “high-performance nanocomposite,” “nanocomposites,” “electrochemical sensor,” “platform,” “sensitive detection,” “surface plasmon resonance,” “amplification” and “immunosensor.”

It can be seen that these words refer both to the substances or materials tested, e.g., non-composite, and to the test methods, e.g., “surface plasmon resonance,” but also to the type of biosensors, e.g., “immunosensor,” “electrochemical biosensor,” or to research questions, e.g., “sensitive biosensor,” “platform.”

Based on the most recent citations of keywords (the above terms are related to the stability of biosensors), we can identify scientific trends and directions of development of a given scientific issue. From the point of view of stability of biosensors, it can be seen that research is growing in the direction of electrochemical biosensors and immunosensors in search of materials that are optimal in terms of durability. The experiments mainly use surface plasmon resonance as a leading research method.

From the point of view of scientific development, international cooperation in the area of development of a particular issue is extremely important. Cooperation between countries, institutions and authors is more likely to lead to progress in related research areas.

**FIGURE 6:**
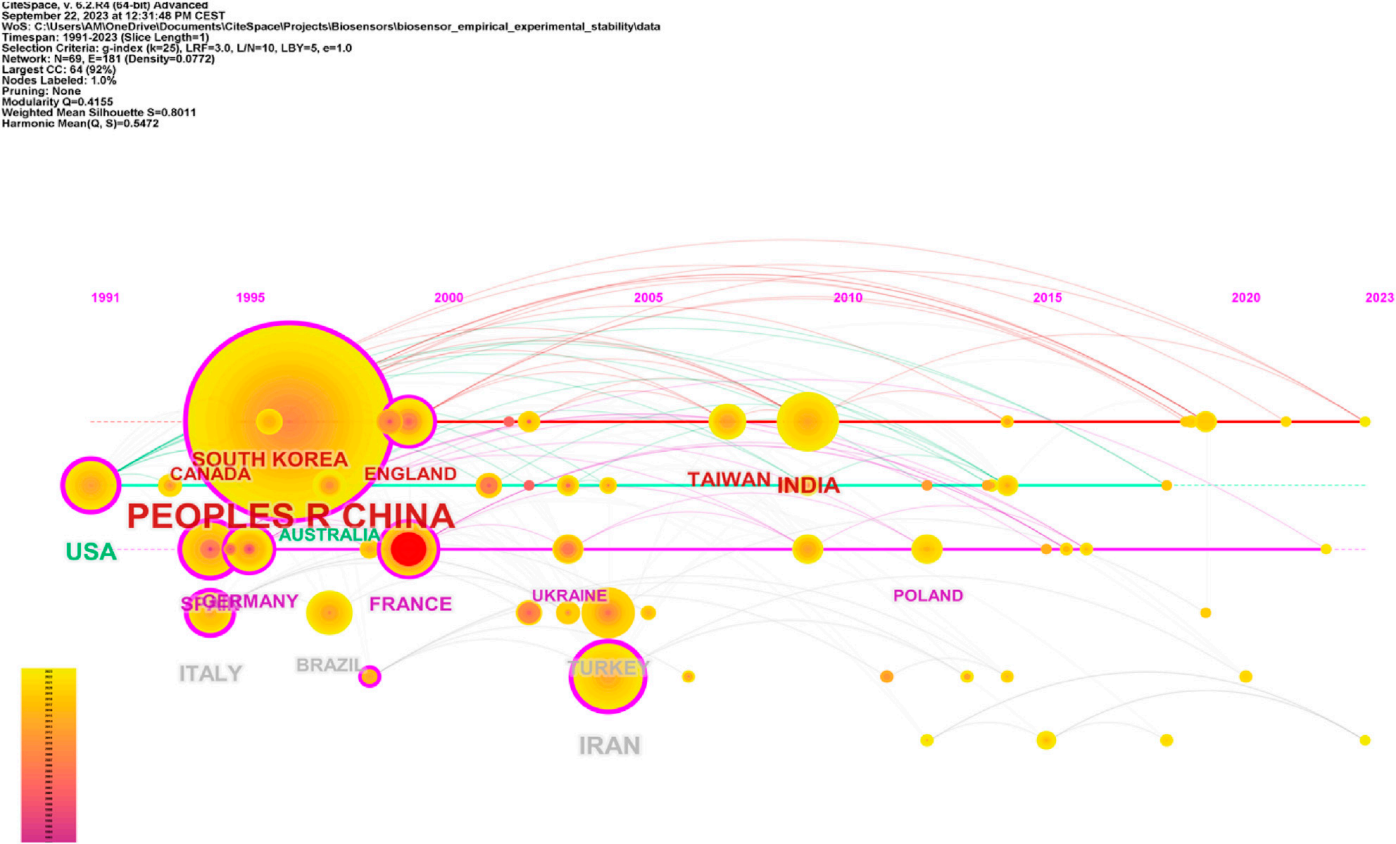
Map of cooperation and networking between countries in the field of biosensor stability.

Figure 6 shows a map of cooperation and networking between countries in the field of biosensor stability. The analysis showed that researchers from 69 countries were working on biosensor stability. The areas marked with circles show the citation rate of representatives of a country (the detailed number of citations of representatives of a country is presented in Table 1). In Figure 6 you can see that the larger the marked area, the higher the citation rate (the graph does not show all countries, but only those whose representatives turned out to be the most active in terms of cited works). Thus, based on the figure below, it can be seen that the most cited works devoted to the stability of biosensors were published by representatives of China (437 citations), but also among the leaders can be distinguished representatives of: Iran (70 citations), United States (57 citations), India (44 citations), Italy (40 citations), South Korea (33 citations), Taiwan (28 citations), Spain (25 citations), France (25 citations), Turkey (24 citations). In 2012, the work of scientists from Poland was also cited.

**TABLE 1:** Number of citations by country representative.

Citation counts	References
437	Peoples R China
70	Iran
57	United States
44	India
40	Italy
33	South Korea
28	Taiwan
25	Spain
25	France
24	Turkey

In the diagram shown in red is the publication of the French representative (as first author) and co-authors (from the United States and Hungary), which generated the widest cooperation in the area of biosensor stability issues. This publication (Theavenot et al., 1999) is extremely important not only in terms of international cooperation, but also in terms of citations and content. The paper is dedicated to the recommendation, classification and definition of electrochemical biosensors. It contains a detailed description of guidelines for reporting biosensor response and calibration characteristics: sensitivity, working and linear concentration ranges, limits of detection and limits of quantification. You will also find information on biosensor selectivity and reliability, response time, reproducibility, stability and device lifetime.

Table 2 presents list of journals in which the most articles related to biosensors have been published. It shows that the highest ranked item in terms of number of citations are the journals: Biosensors and Bioelectronics, Analytical Chemistry, Sensors and Actuators B: Chemical, Analytica Chimica Acta, Talanta and so forward as presented in Table 2.

**TABLE 2:** List of journals in which the most articles related to biosensors have been published.

Citation counts	References
711	Ibiosens Bioelectron
611	Anal Chem
576	Sensor Actuat B-Chem
553	Anal Chim Acta
466	Talanta
372	J Electroanal Chem
361	Electrochim Acta
359	Electroanal
344	J Am Chem Soc
304	Analyst

Comparing the journals in which the most significant articles were published (Figure 4) with the list in which the largest number of articles related to biosensors are found (Table 2), it can be seen that the most cited articles were published in journals: Biosensors and Bioelectronics, Electrochimical Communication, Talanta and Electrochimica Acta. In the table presented (Table 2), the journal Biosensors and Bioelectronics is in the number 1 position, Talanta is in the number 5 position, Electrochimica Acta is in the number 7 position, while Electrochimical Communication is not among the top ten most cited journals.

Thus, it can be seen that there is a kind of correlation between highly cited articles and their impact on the journal’s citability, while the publications with the highest citations do not fully reflect the journal’s cutability.

## Conclusion

This article presents an analysis of the stability of biosensors. This study provides a basic understanding and appreciation of biosensor stability research and shows the direction of scientific development of this topic.

Based on the CiteSpace, v.6.2.R4 tool, an analysis of trends and patterns in the scientific literature was performed. Based on the compilation of keywords, it was shown that the most frequent keywords in the analysed topic are: chemically modified electrode, reduced graphene oxide, direct electron transfer, magnetic microspheres, fft cyclic voltammetry, poly(o-phenylenediamine), lectims, optimization.

This study identified the most important publications in the development of biosensor stability. The 42 most frequently cited words were extracted. Among them, we can distinguish: “graphene oxide,” “high-performance nanocomposite,” “nanocomposites,” “electrochemical sensor,” “platform,” “sensitive detection,” “surface plasmon resonance,” “amplification” and “immunosensor.” From these it can be seen that research is increasing towards electrochemical biosensors and immunosensors. Optimal materials in terms of durability are being sought. The experiments mainly use surface plasmon resonance and fft cyclic voltammetry as leading research methods.

A map of collaborations and transnational networks in biosensor stability showed that researchers from 69 countries were working on biosensor stability. The leaders in citability by country of origin were found to be from China.

Although the Citespace tool has significant potential and greatly facilitates analysis it has certain limitations, inconsistencies and irrelevancies.

CiteSpace analyzes the citations received through the publication of the co-citation network found in the Web of Science bibliographic database.

Web of Science indexes only part of scientific literature. Thus, all analyses, regardless of the methods and tools used, exclude many journals and books.

In the second case, the influence of a specific publication concerns studies whose results are described in publications linked by a parallel citation network, i.e., with smaller or larger thematic links. In addition, it should also be noted that keyword analysis on the basis of titles, abstracts, article descriptions based on articles related to biosensors omits important information contained in books as subsections.

However, despite the limitations of the database, which is the basis of the analysis, CiteSpace remains a very important tool for rapid retrospective and predictive analysis.

## Data Availability

The raw data supporting the conclusion of this article will be made available by the authors, without undue reservation.
